# Assessing cell-nanoparticle interactions by high content imaging of biocompatible iron oxide nanoparticles as potential contrast agents for magnetic resonance imaging

**DOI:** 10.1038/s41598-017-08092-w

**Published:** 2017-08-10

**Authors:** Roxanne Hachani, Martin A. Birchall, Mark W. Lowdell, Georgios Kasparis, Le D. Tung, Bella B. Manshian, Stefaan J. Soenen, Willy Gsell, Uwe Himmelreich, Codi A. Gharagouzloo, Srinivas Sridhar, Nguyen T. K. Thanh

**Affiliations:** 1 Biophysics Group, Department of Physics and Astronomy, University College London, Gower Street, London, WC1E 6BT UK; 2 UCL Healthcare and Biomagnetics and Nanomaterials Laboratory, 21 Albemarle Street, London, W1S 4BS UK; 3 University College London Ear Institute, 332 Gray’s Inn Road, London, WC1X 8EE UK; 4 Department of Haematology, Royal Free Hospital, University College London, London, NW3 2QG UK; 5 MoSAIC/Biomedical MRI Unit, Department of Imaging and Pathology, University of Leuven, B3000 Leuven, Belgium; 6 Gordon Centre for Medical Imaging, Radiology, Massachusetts General Hospital, Harvard Medical School, Boston, Massachusetts USA; 7 Nanomedicine Science and Technology Centre, Northeastern University, Boston, Massachusetts USA; 8 Department of Radiation Oncology, Harvard Medical School, Boston, Massachusetts USA

## Abstract

Stem cell tracking in cellular therapy and regenerative medicine is an urgent need, superparamagnetic iron oxide nanoparticles (IONPs) could be used as contrast agents in magnetic resonance imaging (MRI) that allows visualization of the implanted cells ensuring they reach the desired sites *in vivo*. Herein, we report the study of the interaction of 3,4-dihydroxyhydrocinnamic acid (DHCA) functionalized IONPs that have desirable properties for T_2_ - weighted MRI, with bone marrow-derived primary human mesenchymal stem cells (hMSCs). Using the multiparametric high-content imaging method, we evaluate cell viability, formation of reactive oxygen species, mitochondrial health, as well as cell morphology and determine that the hMSCs are minimally affected after labelling with IONPs. Their cellular uptake is visualized by transmission electron microscopy (TEM) and Prussian Blue staining, and quantified using an iron specific colourimetric method. *In vitro* and *in vivo* studies demonstrate that these IONPs are biocompatible and can produce significant contrast enhancement in T_2_-weighted MRI. Iron oxide nanoparticles are detected *in vivo* as hypointense regions in the liver up to two weeks post injection using 9.4 T MRI. These DHCA functionalized IONPs are promising contrast agents for stem cell tracking by T_2_-weighted MRI as they are biocompatible and show no evidence of cytotoxic effects on hMSCs.

## Introduction

In recent years, research on the development of stem cell therapy has intensified. The potential to use stem cells (SC) in tissue engineering and regenerative medicine is promising, as their use has already been implemented in a few human clinical trials^1–4^. However, a number of questions remain regarding the function of the transplanted SCs as well as their localization and movement. To answer these, certain characteristics of IONPs can be used with a potential of gaining a better understanding of the role of stem cells and validating clinical transplantations^5–9^. Indeed, IONPs may be used to monitor the fate of SCs in a non-invasive manner using MRI. To date, IONPs, which were FDA-approved as MRI contrast agents for the liver have been taken off the market. We have therefore synthesized IONPs for SC tracking by MRI through a high pressure, high temperature method using the polyol route, before functionalizing the surface with 3,4-dihydroxyhydrocinnamic acid (DHCA)^10^. As reported in our previous study, these IONPs-DHCA show great potential as MRI contrast agents as these can be synthesized in a very reproducible manner and their morphology can be finely controlled by the reaction conditions.

A major concern regarding the use of nanomaterials for *in vivo* applications is the potential toxicity they may induce when interacting with biological systems. Most studies have reported the biocompatibility of IONPs with *in vitro* and *in vivo* studies, and it is generally accepted within the scientific community that these are safer materials to use in comparison to other MRI contrast agents, such as gadolinium-based nanomaterials for example^11–13^. However, even though they are widely used and accepted, it is still necessary to assess the toxicity of newly developed IONPs and it is difficult to obtain a consensus amongst researchers on the methods used for determining their toxicity. As highlighted by Paul Weiss *et al*. in ACS Nano editorial in November 2016^14^, the lack of standardization when it comes to the study of nanoparticles remains an obstacle to their potential use in biomedical applications.

To this date, assessment of toxicity is routinely done through colourimetric assays such as MTT or MTS assays, however, it has been demonstrated that there is some interaction of dyes with IONPs and this leads to false positive results^15^. Also, these assays solely report information on a single parameter and on a macroscopic level. Therefore, to circumvent these issues, for this work high-content imaging analysis was used as it provides multiparametric, image - based information on a large number of cells^16^. The large population of cells imaged in an automated manner renders this method quantitative with high statistical power. High content imaging furthermore enables one to measure many parameters at the same time, is able to provide visual confirmation of the results obtained and gives an idea on the variability of any parameter evaluated within the sample tested rather than providing a single unit^17^. This technique allows us to generate large datasets studying various cytotoxicity parameters while experimental conditions are kept consistent for all cellular – IONP interactions. A robust and reliable comparison of potential toxic responses across different conditions can be obtained by this methodology.

In this study, we confirmed the cellular uptake of the DHCA functionalized IONPs by bone marrow-derived primary human mesenchymal stem cells (hMSCs) through direct visualization by Prussian Blue staining and TEM. Their uptake is also quantified by a colourimetric method based on Tiron, which chelates Fe^3+^ and forms a complex which can be measured spectrophotometrically at 480 nm^18, 19^. Finally, *in vivo* visualization of the IONPs was investigated in 6 wk old female Swiss mice by MRI and their accumulation in the liver was visible up to 2 wk post administration. This allowed us to confirm the *in vivo* potential of the IONPs as safe and biocompatible T_2_-weighted MRI contrast agents.

## Results and Discussion

### Synthesis and characterization of IONPs

**Figure 1:**
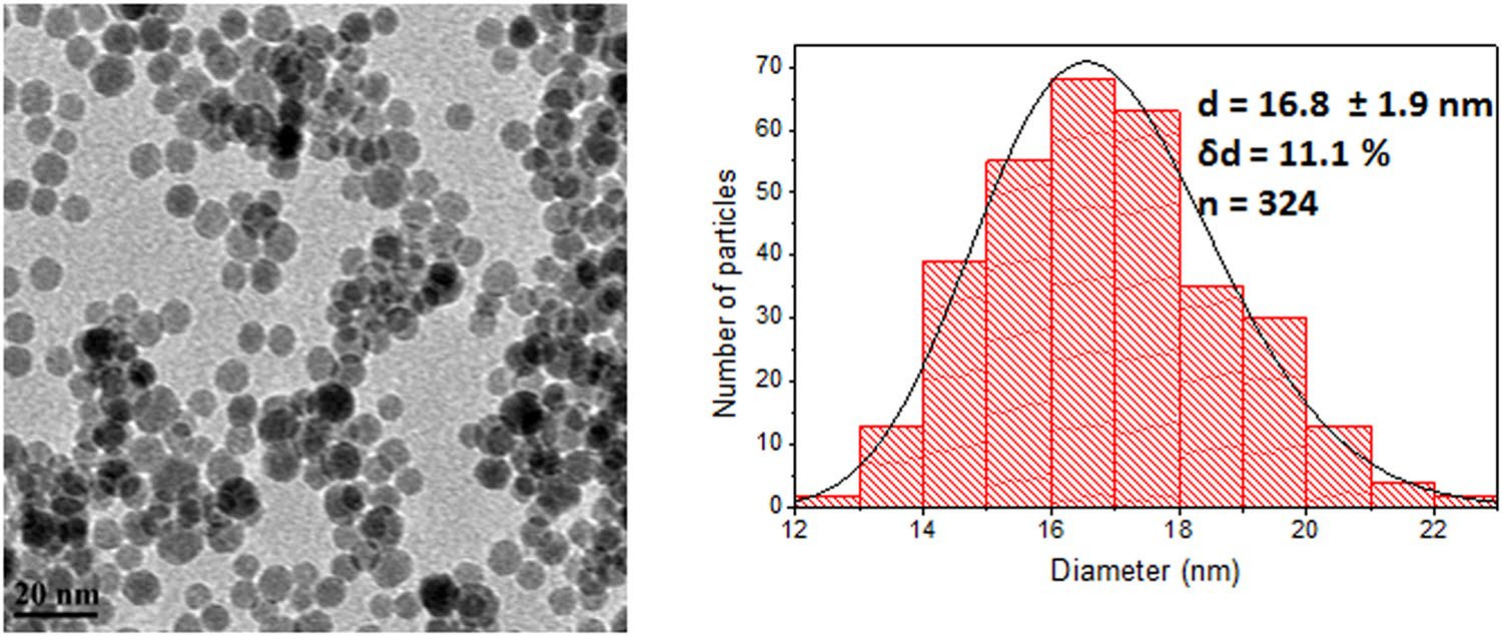
TEM images and particle size distributions of iron oxide nanoparticles synthesized. Magnification 25 k scale bar 20 nm. Size distributions were fitted with a log normal function (solid line), d = mean diameter, δd = standard deviation and n = number of particles counted.

In our previous work, we successfully synthesized IONPs with the surface ligand DHCA^10^. For this study, the IONPs obtained were spherical and with an average diameter of d_TEM_ = 16.8 ± 1.9 nm (δd = 11.1%, n = 324) as determined by TEM (Fig. 1). The hydrodynamic diameter of these IONPs was measured in deionized water by dynamic light scattering (DLS) and was determined to be d_H_ = 88.2 ± 2.4 nm. The zeta potential determined from at least three measurements in water was respectively ζ = −25.5 ± 1.8 mV, at pH = 6.8 and electrical conductance = 0.173 µS. X-ray diffraction was used to confirm that these nanoparticles are indeed iron oxide and have an inverse spinel structure, either magnetite Fe_3_O_4_ or maghemite γ-Fe_2_O_3_, although these phases cannot be distinguished by XRD due to their similar diffraction pattern and peak broadening effects. The IONPs may contain either or both of these iron oxide phases. The crystallite diameter of 7.8 nm determined approximately by the Scherer equation was coherent with that of the core size measured by TEM. The IONPs displayed a superparamagnetic behaviour at room temperature (RT) as measured by SQUID-VSM between −7 and 7 T at 300 K, with a saturation magnetization of M_s_ = 90 emu/g. This value is consistent with superparamagnetic iron oxide nanoparticles of similar size and obtained by the polyol method^10, 20, 21^. This value is slightly smaller than the theoretical magnetization value for bulk magnetite (92–100 emu/g)^22, 23^, and this is due to a finite size effect: canting of surface spins which are unaligned with the spins present in the rest of the magnetic domain^24^. This effect is more pronounced for nanoparticles of smaller size^25^.

### *In vitro* cellular uptake of IONP-DHCA by hMSCs

#### Cell uptake visualized by TEM

**Figure 2:**
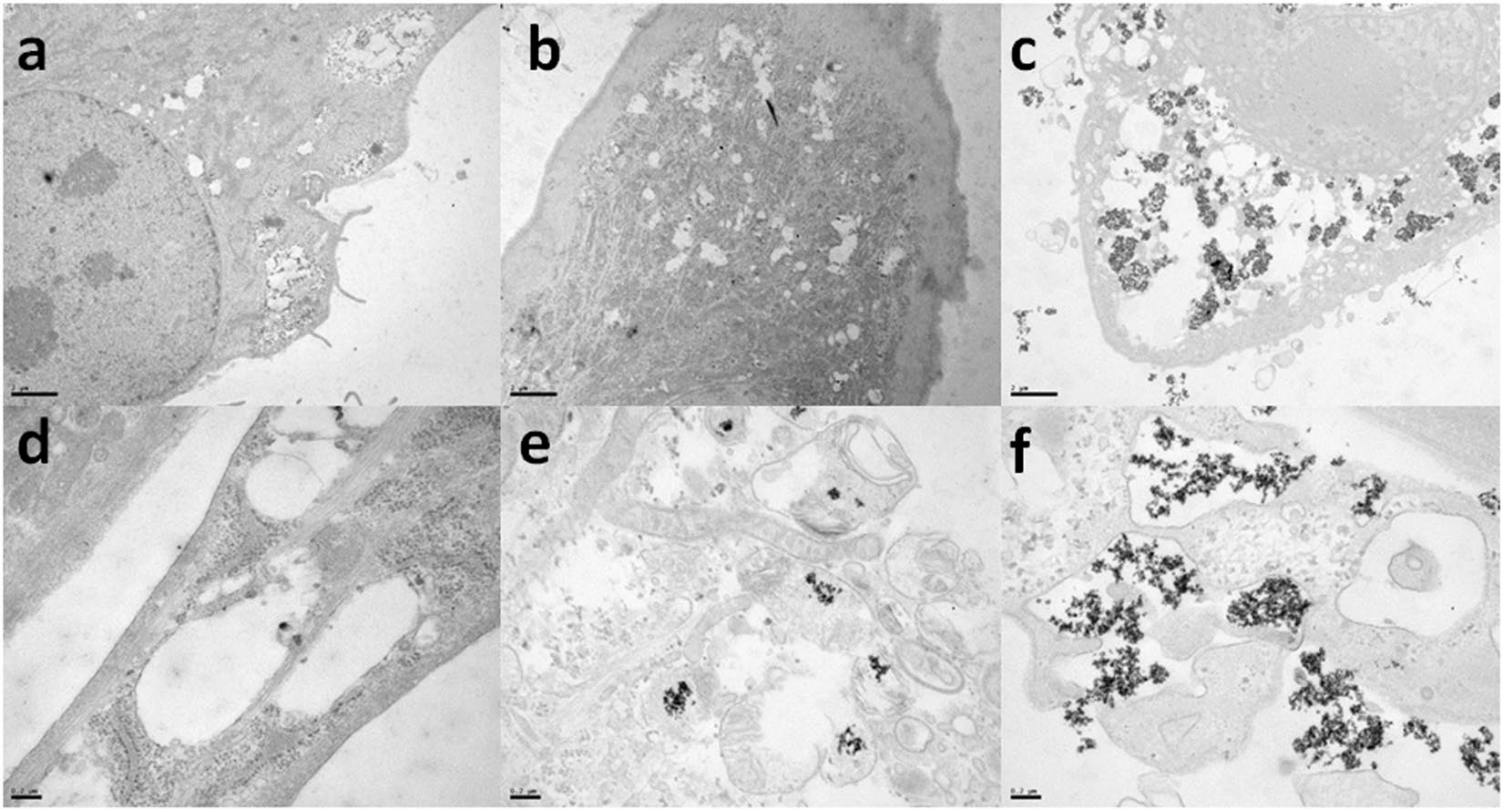
TEM images of hMSCs incubated with IONPs at 50 μg Fe/ml during 1 h (**a**,**d**), 4 h (**b**,**e**) and 24 h (**c**,**f**). Images **a–c** obtained with magnification × 8 k and scale bar = 2 μm and images **d–f** obtained with magnification × 50 k and scale bar = 0.2 μm.

Cell uptake and intracellular IONP distribution in hMSCs were visually confirmed by TEM. We obtained TEM images (Fig. 2), at different incubation times of 1 h, 4 h and 24 h and at a concentration of 50 μg Fe per ml. From the *in vitro* cellular uptake study, it is shown that this concentration is deemed non-toxic and safe where the IONPs did not have any effect on cell morphology, viability, mitochondrial health and did not lead to the production of any reactive oxygen species.

From these images, we can confirm that the uptake of IONPs by hMSCs is successful; however this process is relatively slow as few IONPs are observed after incubation times of 1 h and 4 h. It is only after 24 h that significant amounts of IONPs can be visualized within the cells and at their surface as seen in Fig. 2. As it has also been reported extensively in literature^26–29^, these IONPs seem to undergo endocytosis and can therefore be located in endosomes (Fig. 2f). The IONPs which are internalized within the cell vacuoles could mainly be found as aggregates.

Furthermore, we did not find any IONPs near the nucleus, as it is plausible these aggregates would be physically unable to breach the nuclear membrane pores with sizes in the range of 10–20 nm. Membrane deformation was also observed, confirming the internalization of IONPs by endocytosis.

#### Prussian Blue staining of hMSCs with IONP-DHCA

hMSCs were incubated with IONP-DHCA for 24 h at various concentrations ranging from 0 to 150 μg Fe per ml, then the cells were fixed and stained with Prussian Blue, and the extent of IONP uptake was confirmed by optical microscopy.

The iron-specific Prussian Blue staining (Supplementary Figure S1) also allowed visual confirmation of the association of IONP-DHCA and hMSCs. The labelling efficiency seemed to increase in a dose dependent manner. However, we hypothesize that the uptake is not necessarily more important at the highest concentration, but instead that we observe aggregates of IONPs (examples marked with arrows), which may have attached to the bottom of the well or to the extracellular surface. This may be improved by modifying the IONP surface ligand or the incubation conditions. Overall, it is essential to note that hMSCs incubated with IONP-DHCA remained adherent and maintained their usual fibroblast-like shape similarly to the control.

### Quantification of cell uptake

To quantify the amount of IONPs taken up by hMSCs, we used a colourimetric method based on the chelation of Fe^3+^ by Tiron. The quantity of IONPs taken up by cells is an important factor to consider as this will determine how efficient the nanoparticles are as MRI contrast agents, as well as the impact they will have on the cell viability and proliferation. Below is a table, which sums up the amount of iron taken up by hMSCs and the uptake efficiency (Table 1) after 24 h of incubation. The uptake percentage was determined as the ratio between the final amount of iron measured with 20,000 cells/well, and the initial incubation amount of iron per cell.

**Table 1:** Quantification of cellular uptake of IONPs by hMSCs determined by the colou rimetric method.

Incubation concentration (µg Fe/ml)	Iron uptake per cell determined by colourimetric method (pg)/Uptake percentage (%)
10	72/29
100	819/33
150	1,108/30

These results confirm that the uptake of these IONPs by hMSCs is significant; this can be supported by the TEM images obtained *in vitro* (Fig. 2). The significant uptake in IONPs of hMSCs may be correlated to their strong negative surface charge and is dose dependent. These results tend to confirm that some of the IONPs accounted for may not be internalized, but remain on the surface of the cells. It is a well-established fact that the uptake of IONPs depends on the size of the cells, more precisely their surface area^30^. The surface ligand may contribute to this phenomenon, and may cause IONPs to stick to the bottom of the wells and the surface membrane of cells thus overestimating the amount of iron taken up by cells. We noticed that for high incubation concentrations, as the nanoparticles are in excess in the culture medium, aggregates can be observed (Fig. 2f). Single-cell magnetophoresis is a method which would allow us to obtain accurate and single cell information about the amount of iron taken up by cells. When cells are moving towards a magnet, any IONPs bound to their surface and which have not been internalized, will be visualized as small chains of aggregates transported by the cell. This technique also previously revealed that colourimetric assays can lead to overestimation of the quantity of iron taken up by a factor of three in the case of agglomerated IONPs^31^. Also, flow cytometry is another method used to determine if the nanoparticles are internalized within the cells or not. Nanoparticles must be labelled with a fluorescent dye such as FITC. Trypan blue is a stain routinely used to determine cell viability because it is excluded by viable cells. This characteristic, in addition to the fact that it can quench the fluorescence of FITC^32, 33^, has been used to differentiate between nanoparticles extracellularly associated and those internalized by viable cells^34^. Indeed, their uptake in cells is assessed by flow cytometry with Trypan Blue staining before the measurement. This stain will quench the fluorescence of nanoparticles bound to the cellular surface, whereas the fluorescence of internalized nanoparticles will not be affected^35^.

### Cell – nanoparticle interaction study by MTT and MTS assays

We were able to demonstrate by TEM and Prussian Blue staining that the synthesized IONPs were successfully taken up by endocytosis in hMSCs after 24 h. However, in order to be considered for stem cell labelling applications, we must ensure that these nanoparticles are biocompatible and not toxic to hMSCs when exposed to increasing concentrations of IONPs. For this, we conducted conventional colourimetric assays, MTT and MTS assays, which determine the cytotoxic effects of IONPs on cell metabolic activity or cell viability.

These cytotoxicity assays rely on the same principle and only differ by the nature of the product obtained: the MTS assay does not require solubilization of the formazan compound formed. Initial assays were conducted with hMSCs incubated during 24 h with IONP-DHCA concentrations ranging from 0–1 mg Fe/ml. The results obtained are presented in Supplementary Figure S2.

At lower concentrations of Fe, up to 100 μg Fe/ml, no significant toxic effects were observed by either assay: a cell viability of about 80% was measured at 100 μg Fe/ml. However, it can be observed that the cell viability increases significantly for elevated concentrations of IONPs (500 and 1,000 μg Fe/ml). Supplementary Figure S2c clearly proves that this method is not suitable to assess the cytotoxic effects of IONPs at high concentrations (above 100 µg Fe/ml) as the values measured (Abs > 1) are no longer within the linear absorbance range. The increase in cell viability measured with both assays is therefore due to interference of the IONPs present in solution with the MTT and MTS dye (Supplementary Figure S2). This finding is in line with literature data, where interference of IONPs with the MTT assay has been reported^36, 37^, and demonstrates that colourimetric assays are not technically suitable for high concentrations of IONPs. In most of today’s published research, these assays remain routinely used to confirm the biocompatibility of nanoparticles synthesized with various cell lines^38, 39^. While the interference of IONPs may be deducted from the absorbance values measured, it is not possible to conclude with certainty on the effect of the internalized nanoparticles *in vitro*. Furthermore, these assays only give us average information of the whole cell population being assessed, without being able to directly observe and determine the impact of the nanoparticles on the cells.

High content analysis can be expanded to proliferation assays; however, it is quite difficult to obtain sufficient hMSCs for this extra assay, due to their limited proliferation capacity which is dependent on the age of the donor from which they are sourced and culture conditions (media composition, temperature, CO_2_ and humidity) used for their initial expansion.

### *In vitro* analysis of cell viability by flow cytometry

To assess the effect of IONP-DHCA, and the mode of death they may induce on hMSCs, flow cytometry was tested as it is suitable to determine which cells are viable, apoptotic or necrotic.

Annexin V-PE and 7-AAD double staining was used to detect cell membrane changes as this is routinely used to investigate the effect of nanoparticles on various cell populations. Annexin V binds to phosphatidylserine (PS), which is normally located in the inner cell membrane in healthy cell populations. However, when a cell undergoes apoptosis, phosphatidylserine is flipped and can be found on the external cellular membrane, and can thus bind Annexin V. 7-AAD will bind to DNA, and thus is a marker of necrotic cell death. Unlike propidium iodide (PI)  which is more frequently used than 7-AAD, the fluorescence emitted by 7-AAD has been shown to be more stable and it does not leach from cells^40^.

However, as we can see from the results in Supplementary Figure S3, there are still some difficulties using this method. As it can be seen with the unstained cell population (a), 95% of the cell population is deemed viable (apoptosis negative, necrosis negative) which is expected of a control sample consisting of cells having undergone cell culture, and which is consistent with the Trypan Blue staining conducted (not shown here).

When treating cells with Annexin V and 7-AAD, within the same cell population, only 31% of the cells are viable (apoptosis−, necrosis−), 2% are dead (apoptosis+, necrosis+) and 67% are undergoing apoptosis (apoptosis+, necrosis−). From these experiments, we hypothesized that the abnormally high percentage of cells, which are Annexin V positive are false positives. This is probably due to the detachment of the hMSCs from tissue culture flasks by trypsinisation which may lead to temporary membrane damage, thus leading to an Annexin V positive signal^41, 42^. The method of detachment of cells is cell-line dependent and its effect on the integrity of the cellular membrane cannot be predicted. It is therefore crucial to evaluate the cell detachment method and analytical method to ensure the results obtained are conclusive.

**Figure 3:**
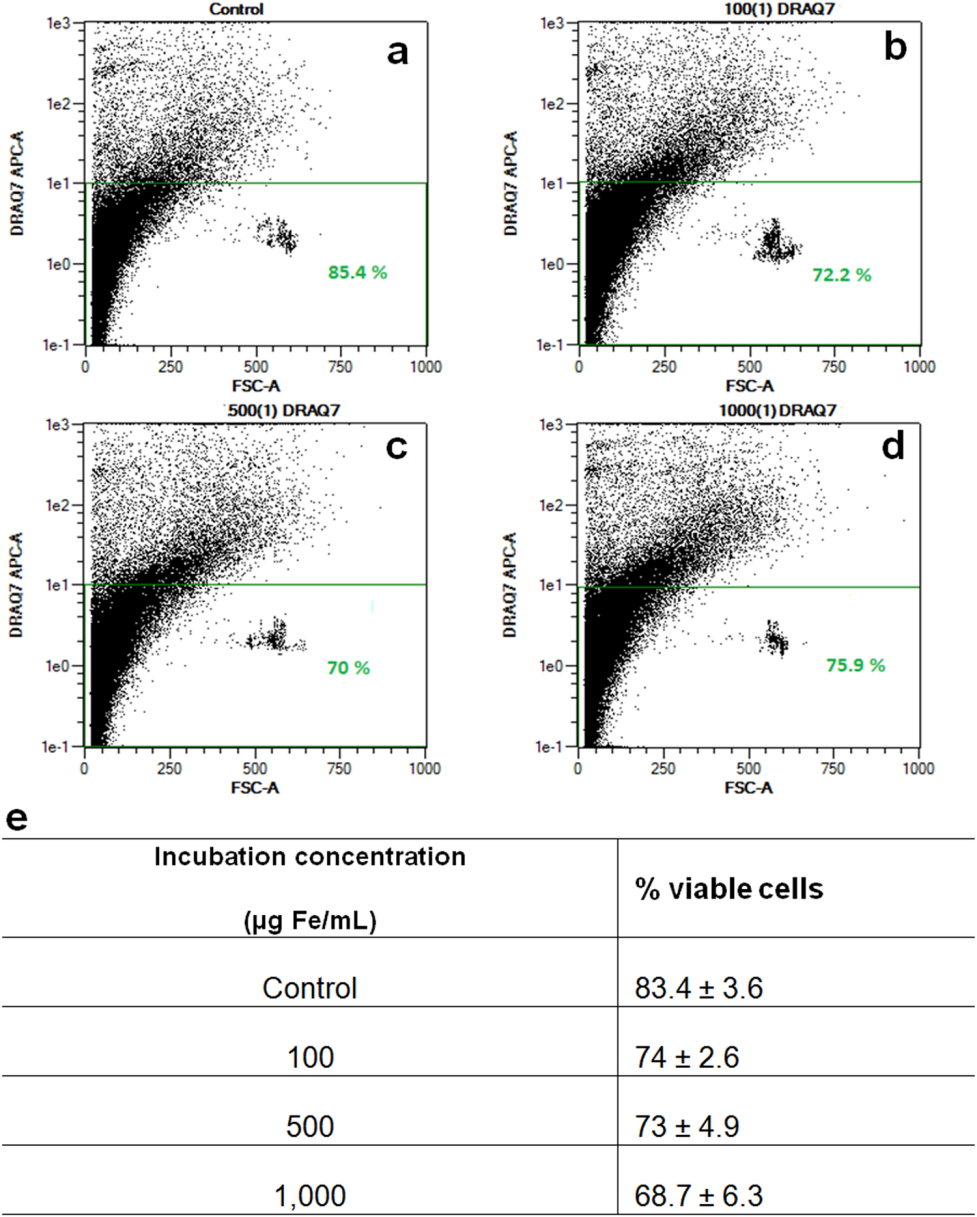
Representative flow cytometry dot-plots obtained for hMSCs after staining with DRAQ7 and incubation during 24 h with (**a**) no IONP-DHCA, (**b**) 100 µg Fe/ml of IONP-DHCA, (**c**) 500 µg Fe/ml of IONP-DHCA and (**d**) 1,000 µg Fe/ml of IONP-DHCA. (**e**) Quantification of the percentage of viable cells determined by DRAQ7 flow cytometry with each condition being done in triplicates.

The flow cytometry analysis was repeated with another stain: DRAQ7, which emits in the far-red region and stains dead cells by binding to the DNA of cells with compromised plasma membranes. This stain did not lead to an abnormally high percentage of dead cells: approximately 85% of cells were deemed viable which is coherent with Trypan Blue staining and 83% of viable cells in the unstained control (Supplementary Figure S3). The results obtained with IONP-DHCA are illustrated in Fig. 3.

After 24 h of incubation with 100 μg Fe/ml of IONP-DHCA, the percentage of viable is 74%, and the corresponding value for 1000 μg Fe/ml is 68.7% (Fig. 3), indicating that the cell death induced in hMSCs by IONP-DHCA is dose-dependent. These results demonstrate that this method is suitable, and DRAQ7 is an adequate stain to determine the percentage of viable cells after exposure to IONPs.

### High content analysis of cell – nanoparticle interactions

**Figure 4:**
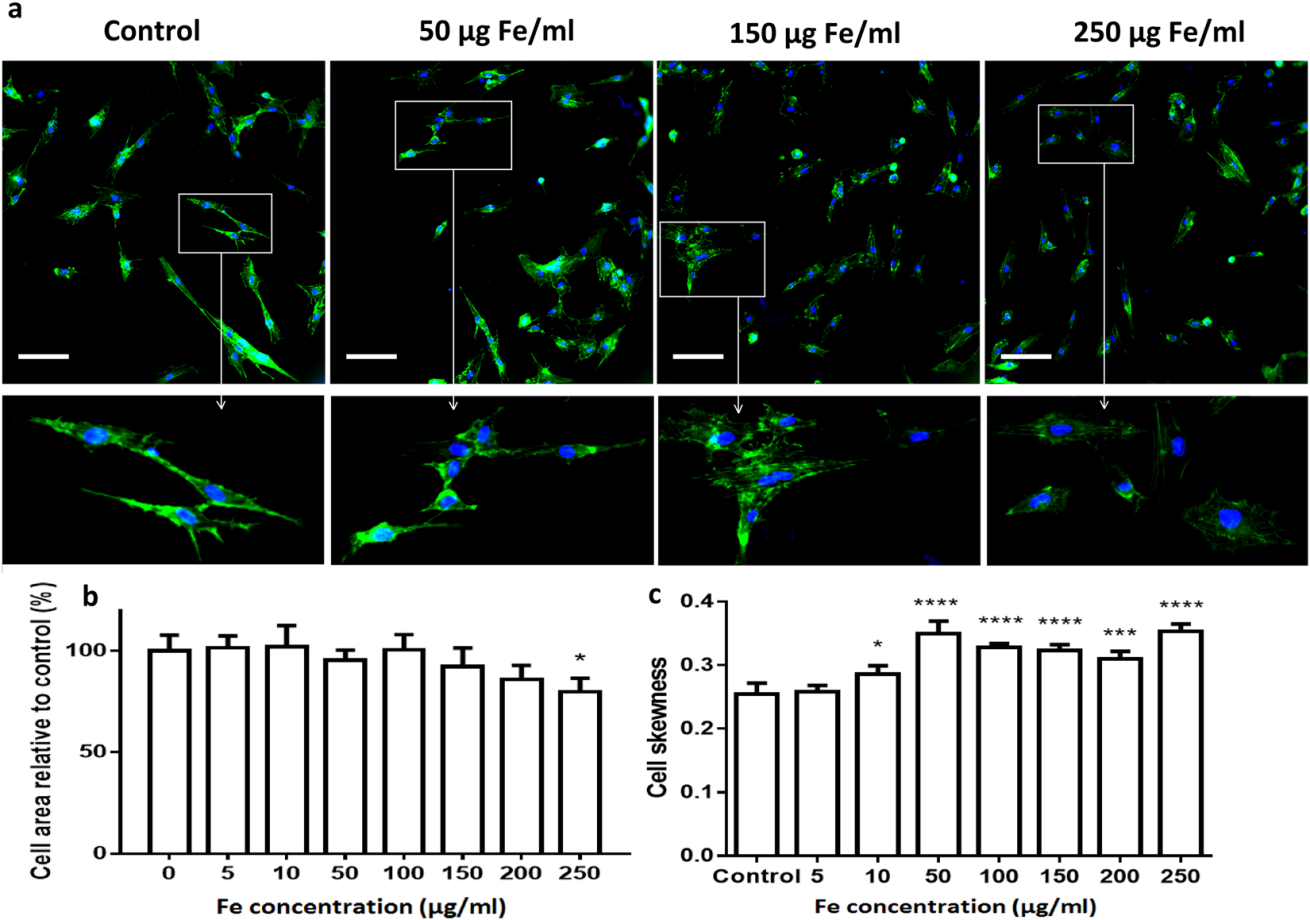
(**a**) Representative images of high-content imaging setup of hMSCs stained for nucleus with Hoechst (blue) and for actin with Acti-stain 48 (green), scale bar 100 µm. From the images captured by high content analysis, the spreading of the cell was calculated (**b**), as well as the cell skewness (width of the cell over the length of the cell) as seen in (**c**). Data are represented relative to untreated control cells as mean ± SD for minimum 500 cells per condition, 3 replicates per condition. The degree of significance is indicated when appropriate *P < 0.05, **P < 0.01, ***P < 0.001 and ****P < 0.0001 (one-way ANOVA, Dunnett post-hoc test).

To overcome the colorimetric interference of IONPs with toxicity assays, high content imaging was used in order to determine cell viability and changes to cellular morphology after exposure to IONP-DHCA. In a 96-well plate, 1,000 hMSCs per well were incubated with IONP-DHCA during 24 h at concentrations ranging from 0 to 250 µg Fe/ml and the nucleus was stained with Hoechst, while actin was stained with Acti-Stain 48 (Fig. 4).

**Figure 5:**
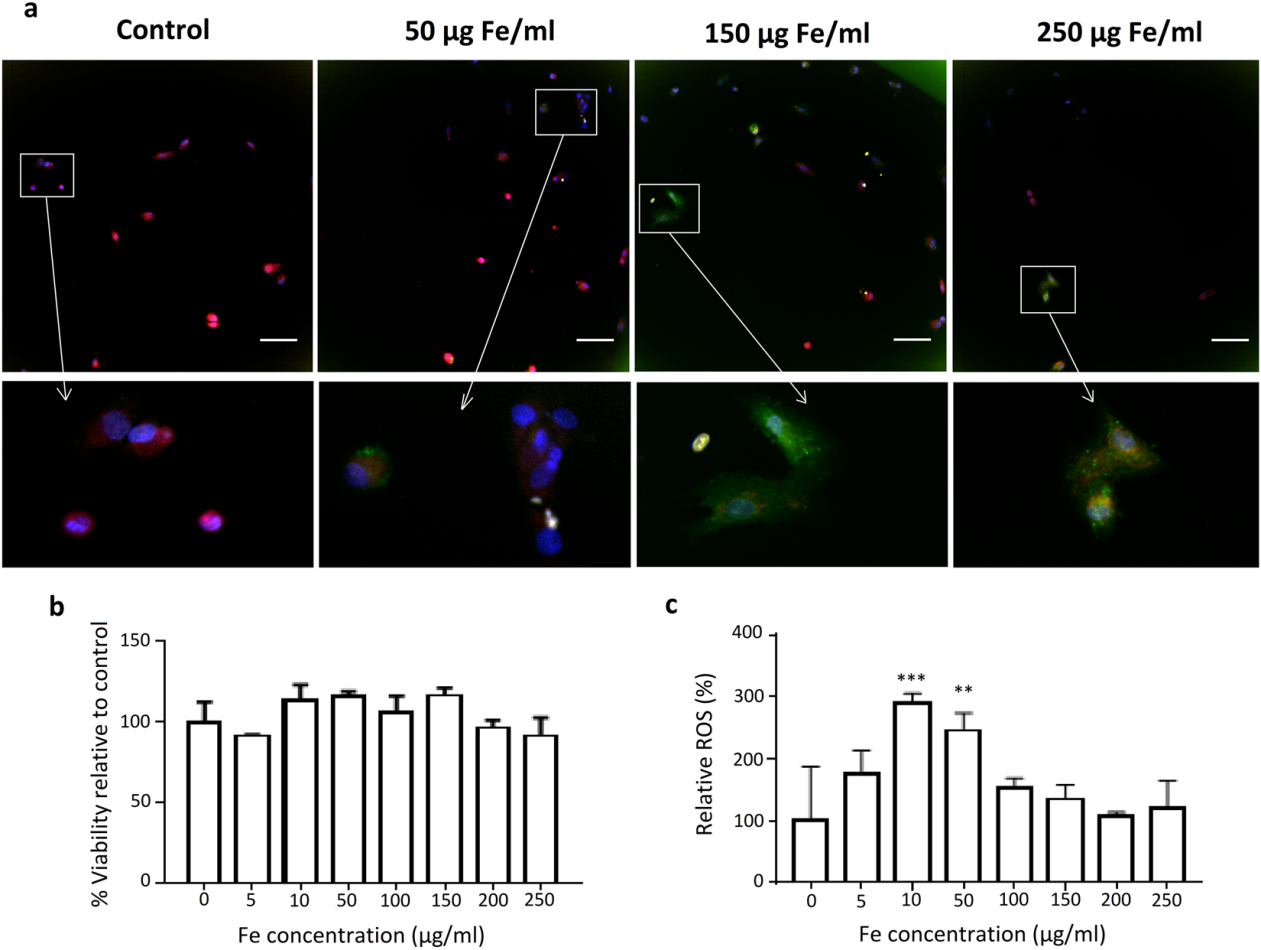
(**a**) Cell viability (yellow), oxidative stress (green) and mitochondrial health (red) of hMSCs labelled with IONPs at various concentrations and determined by high-content imaging reveals significant induction of reactive oxygen species (ROS; green colour) at 10 and 50 µg Fe/ml. Scale bar 100 µm. (**b**) Relative viability and (**c**) production of reactive oxygen species determined by high-content imaging. Data are represented relative to untreated control cells as mean ± SD for minimum 500 cells per condition, scale bar 100 µm. The degree of significance is indicated when appropriate *P < 0.05, **P < 0.01 and ***P < 0.001 (one-way ANOVA, Dunnett post-hoc test).

It has previously been shown that nanoparticles may result in actin cytoskeleton deformation, leading to disruption in actin-mediated cell signalling^43^. In this work, we were able to determine that the cell area was not affected following exposure to low concentrations of IONPs (Fig. 4b) and that it is only above 100 µg Fe/ml that the cell area starts to decrease. However, the IONPs seemed to have a more significant impact on the cell aspect ratio with the cell skewness increasing above 50 µg Fe/ml (Fig. 4c). The latter indicates that the cell length increases as the cells changes from its usual fibroblast-type shape to a slightly more elongated shape. This may be caused by the cells undergoing stress most probably due to the presence of IONPs in the intracellular environment leading to changes in the actin cytoskeleton. Therefore, the cells are unable to stretch as they normally would. The parallel organized thin actin fibres become disordered and lose their original morphology due to the presence of NPs. In order to investigate whether this had an impact on the cellular activity, we then used high-content imaging to determine the effect of the IONPs on cell viability, mitochondrial activity and reactive oxygen species (ROS) formation. The images obtained are shown in Fig. 5a.

The data obtained from high-content imaging are presented in Fig. 5b and c for the relative cell viability and relative ROS formation; and in Supplementary Table S1 for the mitochondrial area and activity. These results clearly demonstrate that the IONPs did not have a significant effect on the viability of the cells or on the mitochondrial area. However, a significant increase in ROS production was noticeable at 10 and 50 µg Fe per mL (Fig. 5c), but this did not have an impact on the mitochondrial health and was not induced at other concentrations (Supplementary Table S1). Elevated ROS production often does not cause any significant toxicity, as all cells possess intrinsic antioxidant properties that protect them against oxidative stress^44^.

### *In vitro* relaxivity of IONP-DHCA

**Figure 6:**
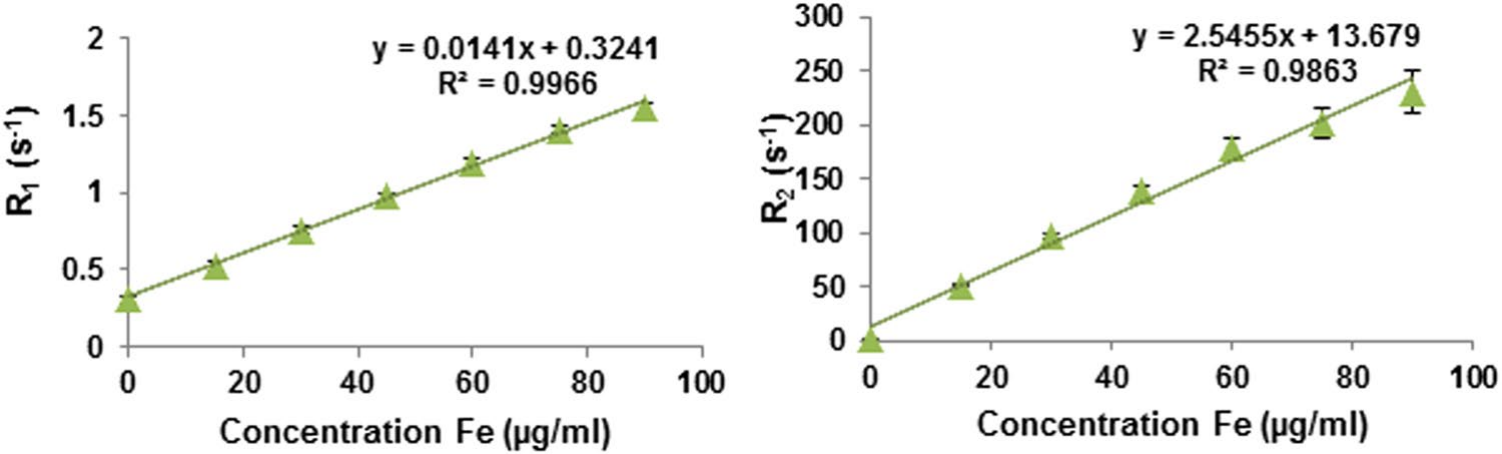
Left plot of relaxation rate R_1_ (R_1_ = 1/T_1_) over Fe concentration of the IONP-DHCA nanoparticles in solution. The slope indicates the specific relaxivity (r_1_); right plot of relaxation rate R_2_ (R_2_ = 1/T_2_) over Fe concentration of the IONP-DHCA nanoparticles in solution. The slope indicates the specific relaxivity (r_2_). Relaxivity values have been converted to mM^−1^ s^−1^ using the molar mass of iron (M = 55.845 g/mol).

To determine the relaxivity value of the IONP dispersion, longitudinal (*T*_1_) and transverse proton relaxation times (*T*_2_) were measured as a function of iron concentration at 7 T, 37 °C. The different concentrations of IONPs for relaxivity characterization were obtained by dilution with deionized water. With these IONP solutions, the observed relaxation rate constant R is linearly dependent on the concentration of Fe. The slope of the dependence is the relaxivity r and the y-intercept is the native relaxation rate of the solution prior to the addition of IONPs. As shown in Fig. 6, the nanoparticles exhibit *r*_1_ and *r*_2_ values of 0.78 and 142.2 mM^−1^ s^−1^, respectively. The *r*_2_/*r*_1_ value of 182.3 confirms that the IONP-DHCA nanoparticle has the potential to be used as a T_2_-weighted MRI contrast agent.

### *In vitro* MR image acquisition

In order to determine the suitability of IONP-DHCA as MRI contrast agents, it is essential to determine their physicochemical properties such as their relaxivity values *in vitro* when they interact with the cells. This will allow us to determine whether any change in relaxation effects occurs once they have been internalized by hMSCs.

Supplementary Figures S5 to S8 clearly demonstrates an overall dose dependent decrease of the mean values of T_1_, T_2_ and T_2_* as a function of the concentration of Fe. This effect has been observed with several types of nanoparticles and has been attributed to their endosomal internalization in cells which causes a clustering and hence an increase in relaxivity^45–47^. Most relevant is the fact that Supplementary Figs S5 to S8 demonstrate that the IONP-DHCA may be used in MRI to provide contrast enhancement.

### *In vivo* MR imaging of IONP-DHCA

**Figure 7:**
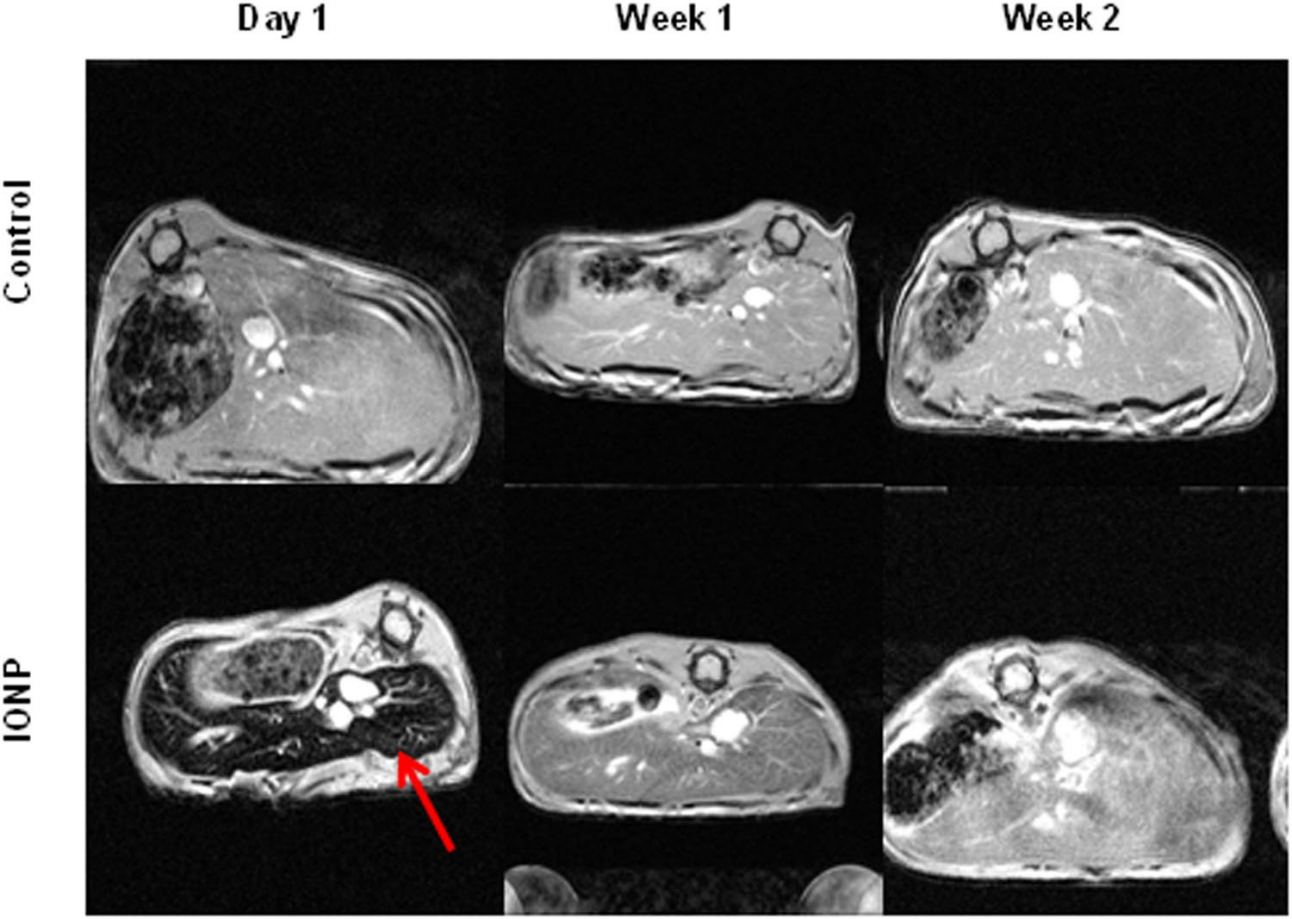
*In vivo* T_2_*- weighted MR imaging showed axial views of mice liver area up to 2 wk after injection, with an area of hypointensity due to the IONP indicated by the arrow. Selected MRI images are representative of three mice that received IONP-DHCA nanoparticles or just PBS (control).

Iron oxide nanoparticles as MRI *T*_2_ / T_2_* contrast agents have been extensively used in liver MRI. Before the animal studies, we first tested the cytotoxicity of IONP-DHCA using hMSCs as a model. The high-content imaging method allowed us to determine that IONP-DHCA have no appreciable cytotoxicity for 24 h even at concentrations up to 250 μg Fe per ml, suggesting the high biocompatibility of these nanoparticles. Furthermore, in our previous work^10^, we determined their potential as MRI contrast agents with their high relaxivity values measured at 1.4 T in solution (r_1_ and r_2_ relaxivities of 7.95 mM^−1^ s^−1^ and 185.58 mM^−1^ s^−1^ respectively). To verify their ability as contrast agents *in vivo*, we conducted *T*_2_-weighted MRI of liver using 6 female Swiss mice as a model. After intravenous injection of IONP-DHCA at a concentration of 300 µg Fe per ml, we immediately observed significant signal attenuation in the liver region for IONP-DHCA (Fig. 7).

**Figure 8:**
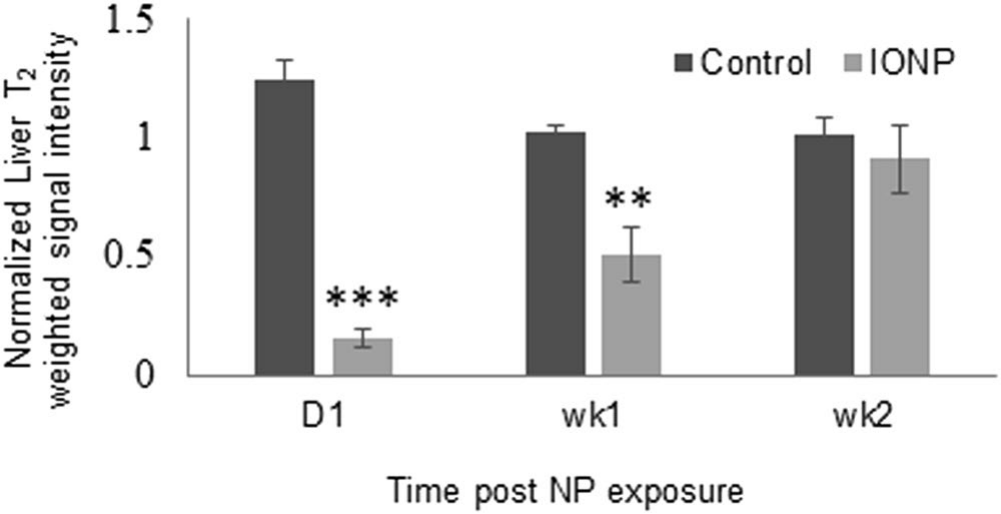
Quantification of the signal intensity of the specific region of interest defined as the liver, as evaluated from the MR images in mice administered PBS (control) and mice administered 60 µg Fe of IONP-DHCA after 1 d, 1 wk and 2 wk post IONP exposure. The signal was normalized against muscle tissue around the vertebra column. The analysis was conducted in replicates of three mice per condition over 2 wk. The degree of significance is measured using GraphPad Prism software and is indicated when appropriate *P < 0.05, **P < 0.01 and ***P < 0.001 (*one*-*way ANOVA*, Dunnett *post-hoc* test).

To quantify the contrast, we identified the liver as the region of interest and calculated the normalized T_2_- weighted signal intensity for each animal over a period of 2 wk. These results are shown in Fig. 8.

As can be expected, we observed the accumulation of IONP-DHCA in the liver, thus leading to a hypointense signal. This was quantified with the measured T_2_ signal in the liver, with a significant decrease in its value immediately post injection and 1 wk later. On the other hand, the control remained relatively constant over the 2 wk period. The MRI signal in the region recovered gradually 2 wk after the injection of the IONP-DHCA nanoparticles, thus indicting that the IONP-DHCA are efficiently cleared from the liver, which is an important condition for the safe use of these NPs in clinical settings, as prolonged retention of IONPs in the liver that are slowly converted to ferroproteins are a serious safety concern^48^.

## Conclusions

This study allowed us to determine the suitability of iron oxide nanoparticles synthesized as potential MRI contrast agents. It provides a comprehensive overview of methods assessing the biocompatibility of IONP-DHCA with hMSCs. We have seen that there remains a lack of standardization in the methods used to determine the impact of IONPs on cells, and these must be considered carefully in order to obtain accurate and reliable data.

We were able to confirm their uptake by hMSCs within 24 h by electron microscopy and iron-specific Prussian Blue staining. This is essential to ensure that the amount of IONPs internalized is sufficient to provide contrast by MRI. We were able ascertain that IONP-DHCA were taken up in large amounts by hMSCs by a colourimetric method. However, we found that the high amounts of internalized IONPs determined suggest some aggregates of IONP-DHCA can be taken up by hMSCs or bound to their surface. This is also supported by the Prussian Blue staining images obtained. As discussed, when quantifying the uptake of IONPs in cells, it is essential to determine whether these nanoparticles are internalized or remain bound to the cellular surface.

Also, an important aspect considered is their toxic effects on the cells. Standard colourimetric MTT and MTS assays were initially used to assess this; but these are not suitable for all IONP concentrations as we observed interference from the IONPs and the data obtained was no longer in the linear absorbance range for the highest concentrations of IONPs (500 and 1,000 µg Fe/ml). To overcome this, multiparametric high-content imaging was used to determine the impact of the IONPs on several factors such as cell viability, mitochondrial activity and cell morphology. No considerable toxic effects were noticed, although slight elongation of the cells could be observed. Furthermore, at 10 and 50 µg Fe/ml, an increase in ROS production was observed but could not be correlated to impaired mitochondria and was limited to these two IONP concentrations.

Finally, the potential of IONP-DHCA as MRI contrast agents was studied *in vitro* and *in vivo*. In solution at 7 T, the nanoparticles had the following relaxivity values: *r*_1_ = 0.78 mM^−1^s^−1^ and *r*_2_ = 142.2 mM^−1^s^−1^. The *r*_2_/*r*_1_ ratio of 182.3 confirms their potential as a T_2_-weighted MRI contrast agent.

Results regarding the safety and properties of nanoparticles may differ between *in vitro* and *in vivo* experiments. After our *in vitro* studies of IONP-DHCA, their *in vivo* administration in Swiss female mice allowed us to confirm that they provide negative contrast by MRI for up to 2 wk post injection in the liver, and they did not induce any visible cytotoxic effects to the mice. These nanoparticles are safely eliminated by renal clearance and provide sufficient T_2_ contrast which can be further optimized by their cell uptake, the latter being dose, incubation concentration or size dependent for example. We have carried out whole body imaging, and we only observed the contrast in the liver. These NPs can therefore be used to pre-label hMSCs in order to visualize this cell population by MRI *in vivo*.

Thus, our study provides new insights into determining nanostructures as biocompatible and efficient MRI contrast agents to label and track stem cells *in vivo*. Moreover, the functionalization of IONPs with antibodies binding to specific cluster of differentiation molecules for example (e.g., CD90), and the limited phagocytic capacity of hMSCs *in vivo* would increase the specificity of the signal. This strategy could include the biofunctionalization of these IONPs towards biological molecules expressed in certain cellular states (for example Caspase 3 with apoptosis) in order to obtain trigger specific information at a cellular level.

hMSCs pre-labelled with IONPs could also be used to detect inflammatory processes as reported in the literature^36, 49, 50^.

## Methods

### Nanoparticle synthesis and characterization

The IONPs were synthesized according to a procedure previously reported^10^. Briefly, 4 mmol of iron acetylacetonate Fe(acac)_3_ (Sigma Aldrich, UK) was dissolved in 20 ml triethylene glycol (TREG) (Sigma Aldrich, UK) and the mixture was transferred into an autoclave vessel. The reaction took place during 8 h at 250 °C before cooling down back to room temperature and the obtained black dispersion was cleaned with acetone by centrifugation at 8,500 rpm 3 times during 10 min. The nanoparticles were dispersed in distilled water and 3,4-DHCA (Sigma Aldrich, UK) was added in a ratio 2:1 to the IONPs in order to displace the surface ligand TREG. The ligand exchange reaction took place at room temperature during 48 h before undergoing dialysis (10 kDa molecular weight cut-off membrane) against distilled water. The water was changed 3 times daily during 5 d until a conductivity of 1 micro Siemens (µS) was measured using a SciQuip Pocket Salinity and conductivity meter (SciQuip Ltd, UK). The hydrodynamic diameter and surface zeta potential were determined in deionized ultrapure water (Milli-Q™ by Millipore) using the Malvern NanoZetaSizer (Laser He–Ne 633 nm, Malvern Instruments Ltd, Worcestershire, UK). The size of the iron oxide core was determined by TEM with at least 300 nanoparticles. The phase composition of the IONPs was determined by X-ray diffraction with a PANalytical XRD using Co Kα (λ = 1.789 Å) radiation from which the crystallite size was approximately calculated using the Scherer equation. The hysteresis loop was recorded at 300 K on a SQUID-VSM with applied magnetic fields between −7 T and 7 T. Finally, the iron concentration was determined by UV absorption according to a previously reported method^52^.

### Cell culture conditions

Bone marrow derived human mesenchymal stem cells (hMSCs) were purchased from Thermo Fisher, UK. hMSCs were cultured in alpha minimum essential media eagle (αMEM) supplemented with 10% foetal bovine serum (FBS). This is defined as the complete medium cMEM. Both reagents were purchased from Life Technologies, UK and used without further modification. All cells were cultured at 37 °C in a humidified atmosphere containing 5% CO_2_.

### Visualization of cellular uptake by TEM

For TEM imaging, cells were harvested by gentle trypsinisation and the cell number was determined with Trypan Blue and a haemocytometer. The cells were then seeded on 12 mm cover slips in 24 well plates and allowed to adhere overnight. The next day, they were rinsed with sterile Hank’s balanced salt solution (HBSS) twice, before incubating them with IONPs dispersed in cMEM, at a concentration previously deemed safe and at which their internalization was observed by TEM (50 µg Fe/ml, V = 500 µl) during different periods of time (1 h, 4 h or 24 h). The media was then removed and the cells were rinsed again with cold sterile HBSS before adding a fixative solution of 2% paraformaldehyde (PFA) and 1.5% glutaraldehyde in 0.1 M PBS pH 7.3 for at least 24 h. The cells were then washed twice with 0.1 M PBS buffer during 5 min. Cells were then post-fixed in a solution of 1% osmium tetroxide and 1.5% potassium ferrocyanine (1 h, 4 °C). Cells were rinsed with 0.1 M PBS, 1% tannic acid in 0.05 M PBS during 5 min, 0.1 M PBS during 5 min and then with dH_2_O during 5 min. The cells were then dehydrated with increasing ethanol (25, 50, 70, 90 and 100%) each during 5 min. Cell layers were infiltrated with increasing embedding medium of epoxy resin (25, 50, 66%) in propylene oxide and transferred into polyethylene capsules. Fresh resin (100%) was added and allowed to harden at 60 °C during 24 h. The resin blocks obtained were sectioned and mounted onto copper TEM grids and examined under a JEOL 1010 TEM at 80 kV.

### Visualization of cellular uptake of IONPs by Prussian Blue staining

hMSCs were seeded on a 96-well-plate (1,000 cells/well) and returned to culture overnight. The visualization of cellular uptake of IONPs by Prussian Blue staining being an image based assay, we aimed for the cells to remain in a monolayer for efficient staining and visualization. Cell loading with nanoparticles dispersed in cMEM was carried out at a range of concentrations from 0 to 250 µg Fe per mL (V = 100 µl), over a period of 24 h. Cells were then rinsed 3 times with HBSS to remove any free IONPs, before fixing with 4% PFA during 15 min at room temperature. The fixative was removed, and then the hMSCs were incubated for 10 min with 2% potassium ferrocyanide and 6% HCl in a volume ratio 1:1, until the appearance of blue colour. The cells were then rinsed with distilled water 3 times leaving the water on the cells for 5 min for each wash. Finally, cells were rinsed twice with HBSS to get rid of any excess stain before being observed and captured using an Olympus BX51 light microscope.

### Quantification of uptake of IONPs by hMSCs by a colourimetric method

This protocol was obtained from a method previously published^53, 57^. This assay being based on absorbance measurements, we aimed to obtain sufficient signal with enough cells in order to differentiate it from background levels. hMSCs were seeded on a 24-well-plate (20,000 cells/well) and returned to culture overnight. Cell loading with nanoparticles dispersed in cMEM was carried out at concentrations 0, 10, 100, and 150 µg Fe per ml (V = 500 µl), over a period of 24 h. Cells were then rinsed 3 times with HBSS to remove any free iron oxide. A standard curve ranging from 0 to 100 μg Fe^3+^ per ml was constructed. After rinsing with HBSS, HCl (9.6 µl, 37%) and HNO_3_ (3.2 µl, 65%) were added to each well, and the volume (28.8 µl) was adjusted with 2-[tris(hydroxymethyl)-methylamino]-ethanesulfonic acid (TES) buffer. Solubilization was enhanced by placing the plate on a shaker at room temperature for 2 h. After shaking, distilled water (52 µl) was added to each well. Then, both the samples and standards were mixed with a 5:1 solution (96 µl) of Tiron (16 μl, 0.25 M) and KOH (80 μl, 4 M), followed immediately by the addition of PBS (160 µl, 0.2 M, pH 9.5). After 15 min, *A*_480nm_ was measured.

### Apoptosis detection by flow cytometry

Cell viability was initially assessed using flow cytometry (MACSQuant Analyser, Miltenyi Biotech, Bergisch Gladbach, Germany) with FITC (fluorescein isothiocyanate) - Annexin V and 7-AAD - Annexin V apoptosis detection kits (BD Biosciences, United Kingdom)^54^. Cells cultured and adherent to tissue culture flasks were trypsinized, counted by Trypan Blue staining and haemocytometer, and resuspended in 1x binding buffer at 10^6^ cells/mL. The 1x buffer is obtained from dilution in water of a 10x concentrate solution. The latter is composed of 0.2 µm sterile filtered 0.1 M HEPES (pH 7.4), 1.4 M NaCl, and 25 mM CaCl_2_ solution. The cell suspension (100 μl, 10^5^ cells) is transferred into a flow cytometry tube and 5 µl of FITC Annexin V and 5 µl of propidium iodide (PI) or 7-AAD is added. The tube is mixed gently and incubated for 15 min at room temperature in the dark. The 1x binding buffer (400 μl) is added to each tube and the cells are maintained on ice and analysed by flow cytometry within 1 h.

To overcome the large number of false positives obtained by Annexin V viability assay by flow cytometry, we aimed to use another method to determine the viability of cells after incubation with different incubation concentrations of IONPs. The far-red fluorescence dye used was DRAQ7 which binds and stains DNA in dead cells only. In comparison to PI, the use of DRAQ7 is advantageous as it does not absorb in the UV range, and is non-toxic to cells so can be used in long term culture of cells for the study of viability of cells by flow cytometry, live imaging or high content screening.

Cells cultured and adherent to tissue culture flasks were trypsinized, counted by Trypan Blue staining and haemocytometer, and resuspended in HBSS at 10^6^ cells/ml. An aliquot of the cell solution is transferred into a flow cytometry tube and DRAQ7 is added to a final concentration of 3 µM (1 µl per 100 µl media). The tube is gently mixed and incubated for 10 min at room temperature in the dark. The cells are maintained on ice and analysed by flow cytometry within 1 h.

### High-content imaging

The high-content (HC) imaging based methodology used here has been demonstrated in various publications to be an effective means for determining interactions between cells and NPs^55, 56^. This technique allows the generation of large datasets comprising of thousands of cells per sample per replicate. Coupled with a sophisticated automated image analysis software, multiple parameters can be studied in a population of cells or at a single cell level.

### Cell viability, oxidative stress and mitochondrial health

hMSCs were seeded in 96-well plates (1,000 cells/well) and were allowed to adhere in a humidified atmosphere. The media was then removed and the cells were incubated with increasing concentrations of IONPs (0, 5, 10, 50, 100, 150, 200 and 250 μg Fe per ml, V = 100 µl) dispersed in cMEM during 24 h. After cell labelling, cells were washed three times with HBSS to remove any remaining free IONPs, after which the cells were incubated with 10 μM 5-(and-6)-chloromethyl-2′,7′-dichlorodihydrofluorescein diacetate, acetyl ester (CM-H_2_DCFDA; Molecular Probes, Invitrogen, Belgium) containing media for 30 min at 37 °C. The dye was then removed and cells were washed with HBSS after which they were exposed to 200 nM MitoTracker Red CMXRos cell containing media and incubated for 30 min at room temperature (RT) in the dark. Cells were then washed and incubated with DRAQ7 3 μM cell media containing 3 µM DRAQ7 during 10 min at RT in the dark. The cells were then fixed with 4% PFA for 15 min. The fixative was washed away, after which the cells were rinsed once with HBSS. The nucleus was then stained with Hoechst 33342 (Thermo Fisher Scientific, Belgium) during 10 min at RT in the dark. The cells were then rinsed with HBSS and the plates were analysed with the InCell analyser 2000, where bright field and fluorescence-based images for the following channels were acquired: DAPI/DAPI (Hoechst nuclear counterstain), FITC/FITC (DCFDA ROS probe), DsRed/DsRed (MitoTracker Red CMXRos) and Cy5/Cy5 (DRAQ7) for a minimum of 500 cells per condition. Data analysis was performed on the InCell Developer software (GE Healthcare Life Sciences, Belgium) using in-house developed protocols. Cell numbers were first determined by counting the number of nuclei. Cell nuclei were segmented based on the DAPI/DAPI channel (Hoechst stain). The level of oxidative stress was based on the FITC/FITC channel. Cell cytoplasm was segmented based on the FITC/FITC channel (autofluorescence), using the DAPI/DAPI channel as seed images. Then, the average intensity of the FITC/FITC channel was measured for every individual cell and normalized to the intensity level of untreated control cells (100%). Mitochondrial health was evaluated similarly using the DsRed/DsRed channel, where the intensity of the MitoTracker Red CMXRos probe depends on the mitochondrial membrane potential and thus is lost in non-functional mitochondria. All red spots localized within a single cytoplasm (based on the FITC/FITC channel) were counted and the average intensity of all mitochondria per cell was then measured. This value was then normalized to the intensity level of untreated control cells (100%). Viability was based on the Cy5/Cy5 channel as DRAQ7 emits at wavelengths above 650 nm. Dead cells were defined as DRAQ7 signal that had an intensity of 3-fold higher than background levels and that co-localized with cell nuclei of the DAPI/DAPI channel. The relative number of dead cells was then determined based on the number of red-stained cell nuclei over the total number of nuclei and normalized to the number of dead cells found for untreated control cells (100%).

### Cell morphology

After cell exposure to the IONPs, cells were washed three times with HBSS and fixed for 15 min at RT with 4% PFA. The fixative was removed, cells were washed once with HBSS after which they were permeabilized with Triton X-100 (1%) for 10 min at RT. Cells were then blocked with 10% serum-containing HBSS for 30 min at RT followed by the addition of 200 µl (1/40 dilution) Acti-Stain 488 (Tebu-Bio, Belgium). Cells were incubated for 30 min at room temperature in the dark. The dye was removed, cells were washed with HBSS and the nucleus was stained with Hoechst 33342 (Thermo Fisher Scientific, Belgium) during 10 min at RT in the dark. The cells were then rinsed before 200 µL fresh HBSS was added to each well and plates were analysed on the InCell analyser 2000, where phase contrast and fluorescence-based images for the DAPI/DAPI and FITC/FITC channels were collected at minimum of 500 cells/condition. Data analysis was performed on the InCell Developer software (GE Healthcare Life Sciences, Belgium) using in-house developed protocols. First, cell nuclei were segmented based on the blue channel (Hoechst stain). Using the green channel, cells were then segmented, where any holes in the cells were filled up and included any cells on the border of the field of view were excluded from the analysis. The segmentation was based on the blue channel as seed channel for the nucleus. The total area of every individual cell was then determined. For determination of skewness (*i.e*. the shape of the cells, being the ratio of cell width over cell length), the same approach was used. After segmentation, the “form factor” was calculated which provides the ratio of the cell width over cell length. This value will always be between 0 (straight line) and 1 (perfect circle).

### Nanoparticles relaxivity in solution

Characterization was performed with MRI. The longitudinal and transverse relaxivities (r_1_ and r_2_ respectively) were measured on a 7 T Bruker Biospec using a quadrature 300 MHz, 30 mm mouse coil (Animal Imaging Research, LLC, Holden, Massachusetts, USA). Additional information with details of all imaging acquisition parameters used can be found in the Supplementary Information.

### *In vitro* MR image acquisition

hMSCs were seeded on a 24-well-plate (10,000 cells/well) and returned to culture overnight. Cell loading with nanoparticles dispersed in cMEM was carried out at a range of concentrations from 0, 1, 5 and 10 µg Fe per ml (V = 500 µl), over a period of 24 h. Cells were then rinsed 3 times with HBSS to remove any free IONPs, before fixing with 4% PFA during 15 min at room temperature. The fixative was removed, and then the hMSCs were stored at 4 °C until use.

Cells were detached from the multiwall plate using cell scrapers (VWR^®^, United Kingdom) and counted on a haemocytometer. Ten thousand cells were retained for each sample, which were aliquoted into a 0.25 ml Eppendorf microfuge tube containing 1.5% agar (Sigma Aldrich, Belgium) in PBS. Samples were mounted onto a phantom holder and stored at 4 °C until ready for MRI scanning. All MRI images were acquired with a 9.4 T Biospec small animal MR scanner (Bruker Biospin, Ettlingen, Germany, horizontal magnet) equipped with actively shielded gradients of 600 mT m^−1^ and using a transmit/receive 72 mm quadrature resonator (Rapid Biomedical, Rimpar, Germany). Additional information with details of all imaging acquisition parameters used can be found in the Supplementary Information (Supplementary Figures S4 to S7).

### *In vivo* magnetic resonance imaging

All experiments involving animals were approved by KU Leuven’s Institutional Animal Care and Use Committee (IACUC; Approval No. P259/2015), in accordance with the principles and procedures outlined in national and European regulations.

All MR images were acquired with the 9.4 T Biospec small animal MR scanner (Bruker Biospin, Ettlingen, Germany, horizontal magnet) described in the previous paragraph using the same set up. Prior to scanning, mice were anaesthetized with 2% isoflurane for induction and 1.5% isoflurane for maintenance (carrier gas 100% O_2_). Three female Swiss mice received PBS only (control), while 3 others received IONPs dispersed in PBS through intravenous injection of 200 µl of 300 µg Fe /ml (42.3 µmol Fe/kg) diluted in PBS. Animals were scanned on the day of the injection, then once a week for the next 2 wk. The *in vivo* MR imaging protocol used for liver imaging consisted of 2D T_2_^*^-weighted fast low angle shot (FLASH) and a multi-slice-multi-echo (MSME) sequence. The FLASH sequence (TE = 2.3 ms, TR = 203 ms, flip angle = 30 degrees, FOV = 30 × 30 mm, matrix = 256 × 256, 9 contiguous axial slices of 1 mm thickness acquired in an interleaved scheme, averages = 10) was used to determine the decrease in the signal intensity (SI) post injection. T_2_ values (maps) were determined from the MSME experiments and were used for a semi-quantitative analysis. Parameters for the MSME sequences were TR of at least 3,000 ms, echo spacing of 7 ms, with 234 mm² in plane resolution with six slices of thickness 1 mm each. In order to evaluate particle distribution post intravenous administration in other organs, mice were subjected to whole body scan with a Rapid Acquisition with Relaxation Enhancement (RARE) sequence (TE = 15.88 ms, TR = 6,000 ms, spatial resolution of 200 mm², slice thickness = 0.5 mm with 50 slices) was performed. Mice were monitored using a monitoring and gating model (type 1030) from Small Animal Instruments Inc. (SAII, Stony Brook, NY, USA) for controlling physiological parameters. Temperature and respiration were monitored throughout the experiment and maintained at 37 °C and 40 to 100 breaths min^−1^.

## Electronic supplementary material

Supplementary information
