# Supplementary material for: Assessing cell-nanoparticle interactions by high content imaging of biocompatible iron oxide nanoparticles as potential contrast agents for magnetic resonance imaging
Source: Sci Rep. 2017 Aug 10;7:7850. doi: 10.1038/s41598-017-08092-w (PMC5552868; doi:10.1038/s41598-017-08092-w)
Supplement: Supplementary file 1 — Supplementary information [file 41598_2017_8092_MOESM1_ESM.pdf]

Supplementary information

Assessing cell-nanoparticle interactions by high content imaging of biocompatible iron oxide nanoparticles as potential contrast agents for magnetic resonance imaging

Roxanne Hachani[1,2], Martin A. Birchall[3], Mark W. Lowdell[4], Georgios Kasparis[1,2], Le D. Tung[1,2], Bella B. Manshian[5], Stefaan J. Soenen[5], Willy Gsell[5], Uwe Himmelreich[5], Codi A. Gharagouzloo[6,7], Srinivas Sridhar[6,7], Nguyễn T. K. Thanh[1,2,*]

[1]Biophysics Group, Department of Physics and Astronomy, University College London, Gower Street, London WC1E 6BT, UK

[2]UCL Healthcare and Biomagnetics and Nanomaterials Laboratory, 21 Albemarle Street, London W1S 4BS, UK

[3]University College London Ear Institute, 332 Gray's Inn Road, London WC1X 8EE, UK

[4]Department of Haematology, Royal Free Hospital, University College London, London NW3 2QG, UK

[5]MoSAIC/Biomedical NMR Unit, Department of Medicine, Catholic University of Leuven, B3000 Leuven, Belgium

[6]Nanomedicine Science and Technology Centre, Northeastern University, Boston, Massachusetts, USA

[7]Department of Radiation Oncology, Harvard Medical School, Boston, Massachusetts, USA

***** Corresponding Author
E-mail: ntk.thanh@ucl.ac.uk; Fax: +44 (0)207 670 2920; Tel: +44 (0)207 491 6509

Materials and Methods

Nanoparticles relaxivity in solution

Six 2-ml vials containing the diluted solutions were arranged in a circular pattern around a blank vial of distilled water for the IONP formulation. r_1 was measured with a variable TR spin-echo sequence and r_2 was measured with a multi-echo spin-echo sequence with TR fixed. ParaVison 5.1 software was used to draw regions of interest (ROIs) and calculate relaxation rate values. Decay curves were fit with a mono-exponential decay equation to calculate T_2, $y = A + Ce^{-TE/T_2}$ and a mono-exponential growth saturation recovery equation to calculate T_1, $y = A + C\left(1 - e^{-TR/T_1}\right)$, where A is the absolute bias and C is the signal intensity T_2 of the samples was measured using the Bruker Multi-Slice Multi-Echo (MSME) pulse sequence with the following parameters: Effective TE: 4.25 ms, with 30 echoes ranging 4.25-127.5 ms, TR = 2,500 ms, 40x40 mm^2 x-y field of view (FOV), 30 mm slice thickness, 200 × 200 matrix size providing 200 μm^2 x-y resolution, number of averages = 2, total acquisition time

= 11 m 55 s.

T_1 of the samples was measured using a Rapid Acquisition with Refocused Echoes variable TR (RARE VTR) pulse sequence with the following parameters: TE = 4.25 ms, Rare Factor: 1, 8 TRs ranging from 100 – 2,500 ms, 40 x 40 mm^2 x-y field of view (FOV), 30 mm slice thickness, 200 × 200 matrix size providing 200 μm^2 x-y resolution, number of averages = 1, total acquisition time = 27 m 15 s. For DI-$H_2$0 T_1 & T_2, two separate scans were run with effective TE = 40 ms for T_2 and TRs ranging from 100 – 3,500 for T_1.

Cell – nanoparticle interaction studies

MTT assay

The MTT colorimetric assay reflects the ability of viable cells to convert the soluble MTT [3-(4,5-dimethylthiazol-2-yl)-2,5-diphenyltetrazolium salt (yellow colour) into an insoluble formazan precipitate (purple colour). These reactions are the result of the succinate dehydrogenase enzyme's activity, which is part of the mitochondrial electron transport system. The precipitate obtained is dissolved in organic solvents and its concentration can be determined by spectrophotometry. The amount of the formazan dye generated is directly proportional to the number of metabolically active cells.

hMSCs were seeded on a 96-well-plate (10,000 cells/ well) and returned to culture overnight. Cell loading with nanoparticles was carried out at concentrations from 0 to 1 mg Fe per mL, over a period of 24 h. Cells were then rinsed 3 times with HBSS to remove any free IONPs. MTT was dissolved at a concentration of 5 mg/mL in HBSS and sterile-filtered. Cells were then put in presence of 190 µL of HBSS and 10 µL of the MTT solution. After 4 h, the solution was removed and 200 µL of DMSO or acidic isopropanol (0.04-0.1 M HCl in absolute isopropanol) was added to each well. After 10 min of shaking, A_{570nm} was measured and the background was read at A_{620nm} using BMG FluoStar Galaxy Optima Microplate Reader (BMG LabTech GmbH).

MTS assay

Similarly, the MTS assay is based on the reduction of the tetrazolium salt MTS (3-(4,5-dimethylthiazol-2-yl)-5-(3-carboxymethoxyphenyl)-2-(4-sulfophenyl)-2H-tetrazolium) and an electron coupling reagent PES (phenyl ethosulfate) into a soluble formazan product. hMSCs were seeded on a 96-well-plate (10,000 cells/ well) and returned to culture overnight. Cell loading with nanoparticles was carried out at concentrations of Fe from 0 to 1 mg/ml, over a period of 24 h. Sterile-filtered MTS solution (20 µl) was then added to each well. After 4 h of incubation, the absorbance was read at 492 nm using BMG FluoStar Galaxy Optima Microplate Reader (BMG LabTech GmbH).

Results and Discussion

In vitro Prussian Blue staining of hMSCs with IONP-DHCA

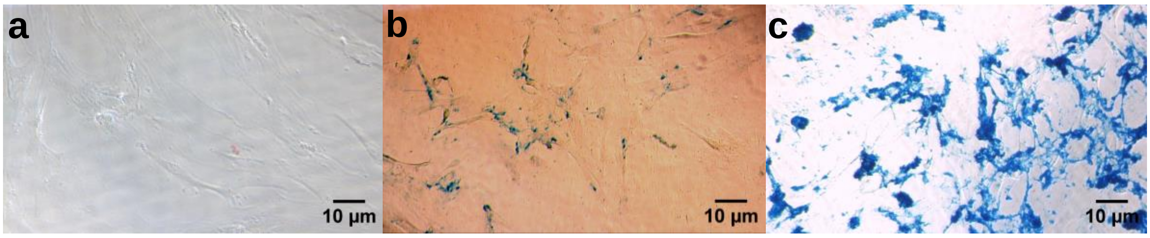

Supplementary Figure S1 Prussian Blue staining images of cells labelled for 24 h with a) 0, b) 50, and c) 150 µg Fe per mL of IONP-DHCA. Scale bar 10 µm.

Cell – nanoparticle interaction studies

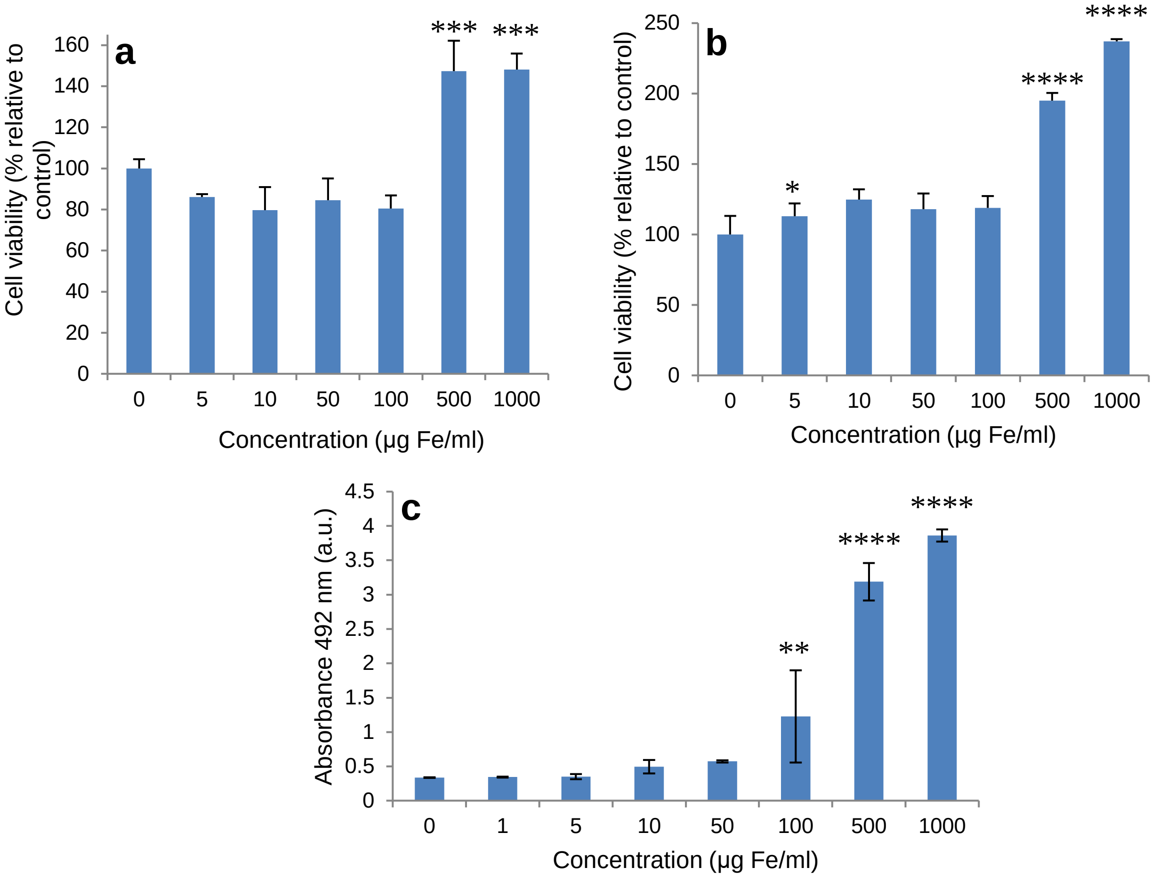

Supplementary Figure S2 *In vitro* cell viability studies on human mesenchymal stem cells after 24 h incubation with IONP-DHCA at concentrations ranging from 0 to 1,000 µg Fe per mL. a) MTT assay and b) MTS assay. c) Absorbance of IONP-DHCA only at different Fe concentrations, measured at 492 nm in the same conditions as MTT and MTS assays performed on hMSCs. Each graph shows the mean + SD (standard deviation) of three independent experiments. The degree of significance is indicated when appropriate $*P < 0.05$, $**P < 0.01$, $***P < 0.001$ and $****P < 0.0001$ (*one-way ANOVA*, Dunnett post-hoc test).

Flow cytometry analysis with Annexin V-PE 7-AAD double staining

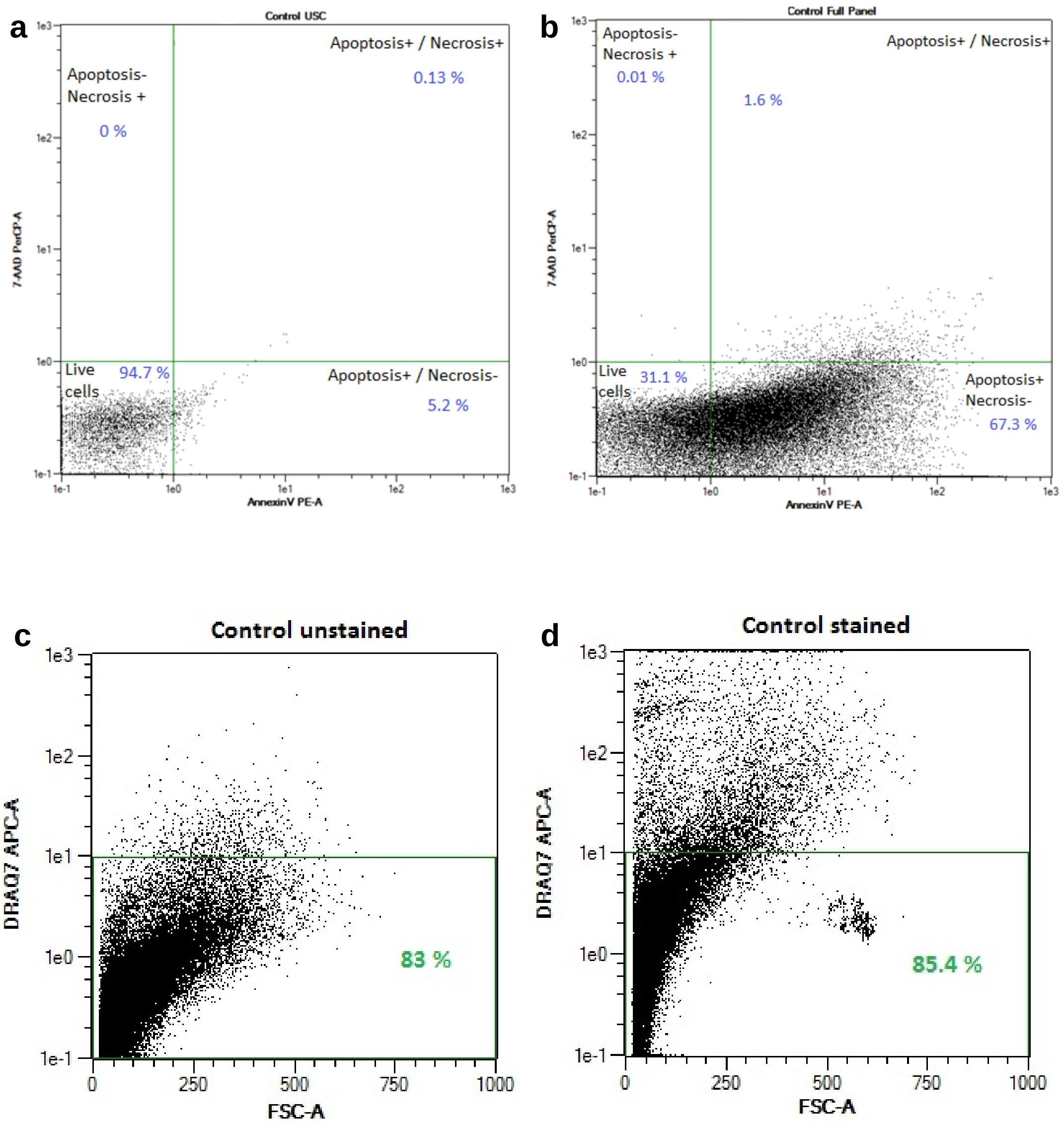

Supplementary Figure S3 Representative flow cytometry dot-plots obtained of a control sample consisting of only hMSCs. a) Before and b) after staining cells with Annexin V-phycoerythrin (x-axis) and 7- Aminactinomycin D (y-axis); c) Before and d) after staining cells with DRAQ7 (y-axis)

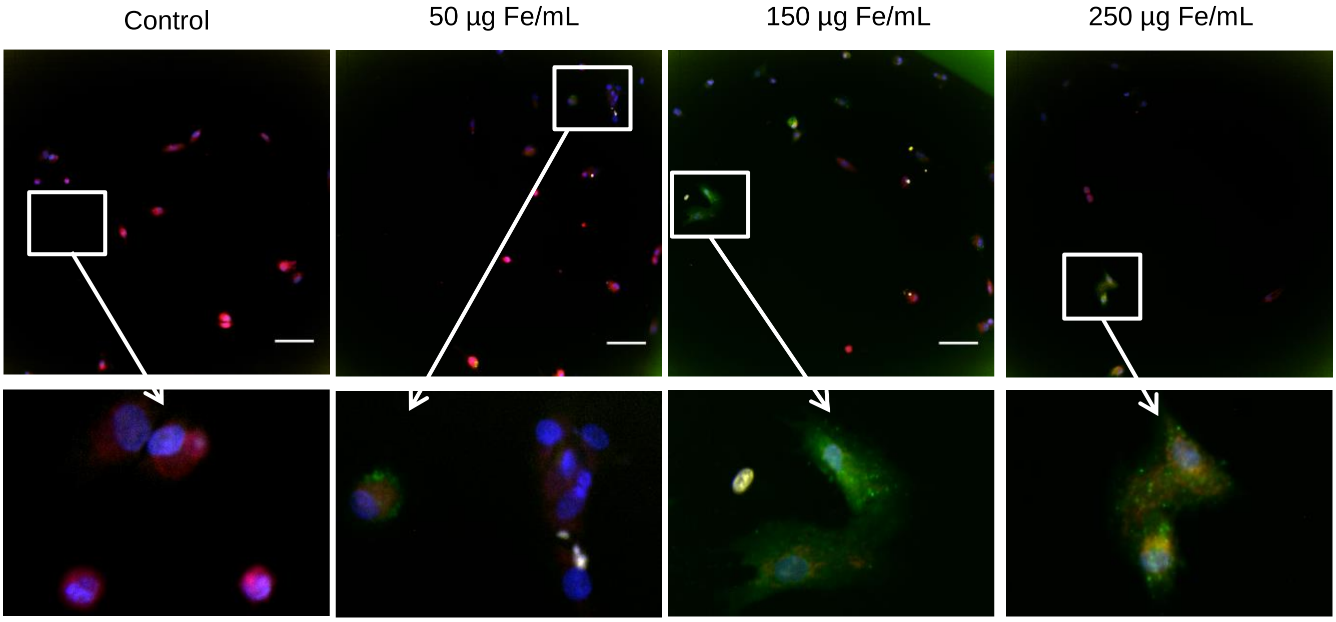

Supplementary Figure S4 Cell viability (yellow), oxidative stress (green) and mitochondrial health (red) of hMSCs labeled with IONPs at various concentrations and determined by high-content imaging reveals significant induction of reactive oxygen species (ROS; green colour) at 10 and 50 µg Fe/ml. Additionally, these particles display reduction in mitochondrial viability (red colour) for a high concentration of 250 µg Fe/ml.

Supplementary Table S1 From the images captured by high content analysis, mitochondrial area and mitochondrial activity were calculated. Data are represented relative to untreated control cells as mean ± SD for minimum 500 cells per condition. The degree of significance is indicated when appropriate *P < 0.05, **P < 0.01 and ***P < 0.001 (one-way ANOVA, Dunnett post-hoc test).

**Fe concentration (μg/mL)**	**Relative mitochondrial area (%)**	**Relative mitochondrial activity (%)**
0	100 ± 7.6	100 ± 19
5	89 ± 19.9	88.9 ± 8
10	91 ± 18.9	84.6 ± 1.6
50	75.7 ± 14.9	91.3 ± 8.2
100	85.8 ± 20.9	83.6 ± 5.4
150	88.1 ± 5.9	83.2 ± 6.4
200	86.6 ± 4.9	80.4 ± 3.7
250	75 ± 18.7	77.1 ± 2 *

***In vitro* MR image acquisition**

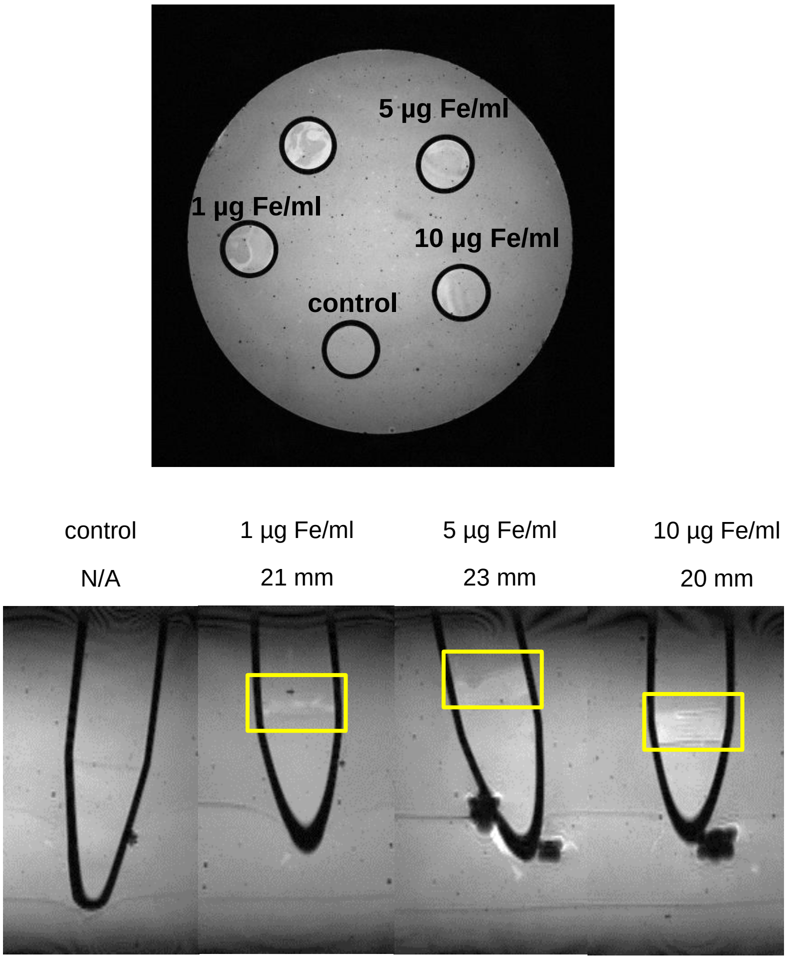

Supplementary Figure S5 Phantom homogeneity and positioning was assessed using FLASH 3D sequence (TE/TR: 15/200ms, matrix: 512x512x128, FOV: 60x60x30 mm, resolution: 0,117x0,117x0,234 mm). A: axial view of the phantom with samples containing 0, 1, 5 and 10 µg Fe/ml. Distance from the bottom of the phantom for each sample being respectively 21, 23, and 20 and 21 mm.

T_1 measurements

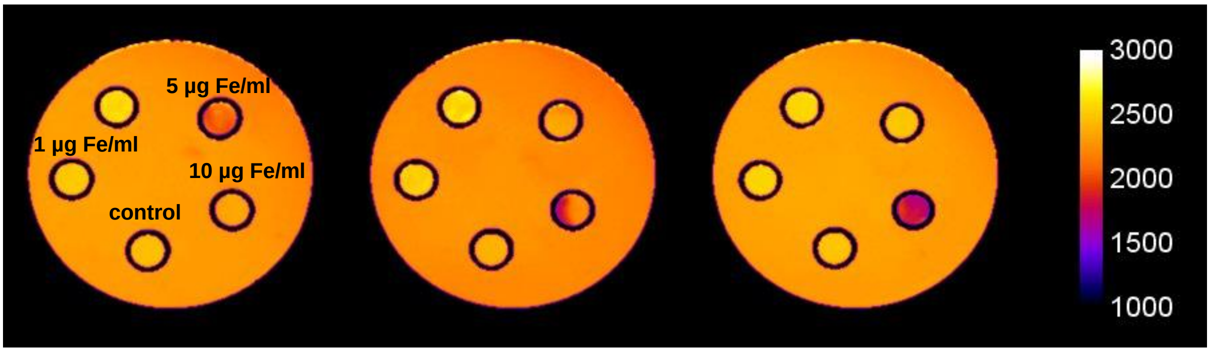

**Fe concentration (µg/mL)**	**T_1 mean (ms)**
0	2,388 ± 19
1	2,515 ± 27
5	2,071 ± 60
10	1,788 ± 92

Supplementary Figure S6 Data were extracted from slices 2, 2, 1 and 3 for samples containing 0, 1, 5 and 10 µg Fe/ml respectively. Spin echo sequence with inversion preparation (TR: 10,000 ms, TE: 5.67 ms, TI: array of 12 Tis starting from 50 ms with regular increment of 500 ms, FOV: 60 x 60 mm, matrix: 256 x 256, 5 slices of 1 mm thickness and 1 mm gap). Data were fitted to a mono-exponential recovery function and T_1 extracted (processing performed in PV5,1 using image sequence analysis tool.

T_2 measurements

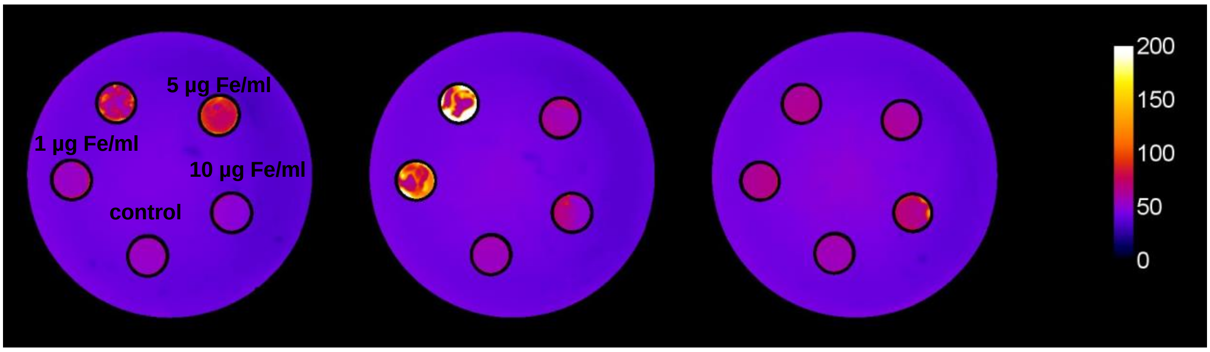

Fe concentration (µg/mL)	T_2 mean (ms)
0	54.5 ± 0.9
1	101 ± 29.9
5	80.9 ± 7.5
10	70.8 ± 10.8

Supplementary Figure S7 Data were extracted from slices 2, 2, 1 and 3 for samples containing 0, 1, 5 and 10 µg Fe/ml respectively. Multi Spin Echo sequence (TR: 5,000 ms, TE: 20 TEs equally spaced by 11.35 ms, FOV: 60 x 60 mm, matrix: 512 x 512, 5 slices of 1 mm thickness and 1 mm gap). Data were fitted to a mono-exponential and T_2 extracted (processing performed in ImageJ using the MRI processor plugin. T_2 maps were generated for pixels for which the R^2 of the fit was higher than 0.9 and capped at 1,000 ms)

T_2* measurements

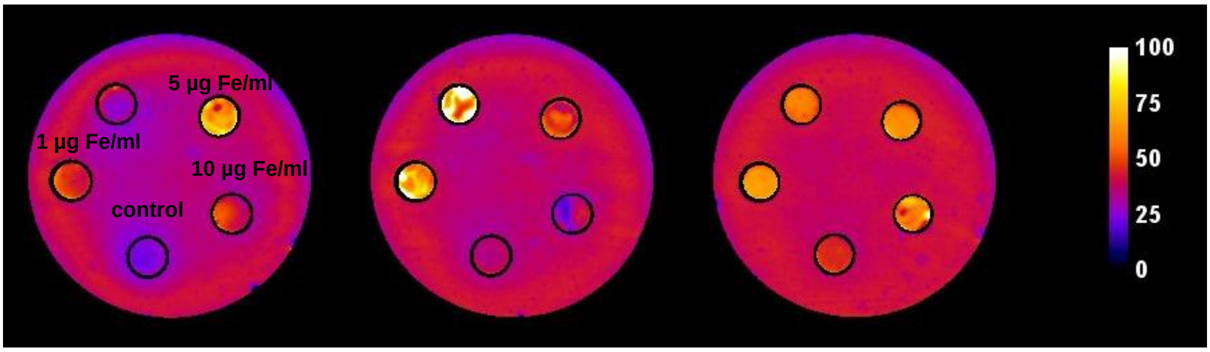

Fe concentration (µg/mL)	T_2* mean (ms)
0	35.2 ± 1.6
1	71.4 ± 17.4
5	64.5 ± 9.1
10	59.5 ± 10.7

Supplementary Figure S8 Data were extracted from slices 2, 2, 1 and 3 for samples containing 0, 1, 5 and 10 µg Fe/ml respectively. Multi gradient echo sequence (TR: 5,000 ms, TE: 14 Tes starting from 2.5 ms with regular spacing of 4 ms, FOV: 60 x 60 mm, matrix: 256 x 256, 5 slices of 1 mm thickness and 1 mm gap). Data were fitted to a mono-exponential and T_2* extracted. Processing performed in ImageJ using the MRI processor plugin. T_2* maps were generated for pixels for which the R^2 of the fit was higher than 0.9 and capped at 200 ms)

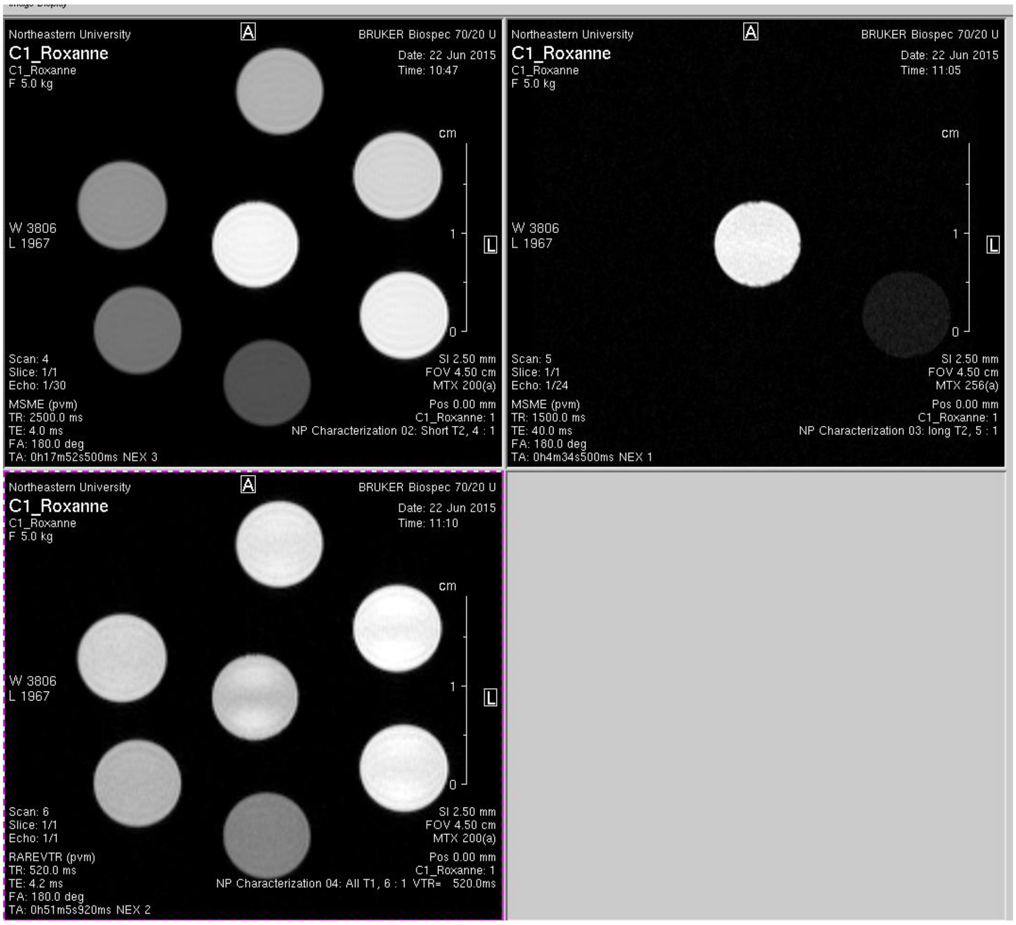

Supplementary Figure S9 r_1 and r_2 maps of IONP-DHCA in solution that were generated at 7 T
